# Human epidermal growth factor receptor 2-positive metastatic breast cancer with novel epidermal growth factor receptor *-ZNF880* fusion and epidermal growth factor receptor E114K mutations effectively treated with pyrotinib: A case report

**DOI:** 10.1097/MD.0000000000023406

**Published:** 2020-12-18

**Authors:** Li Yue, Liu Wentao, Zhang Xin, Huang Jingjing, Zhang Xiaoyan, Fu Na, Ma Tonghui, Li Dalin

**Affiliations:** a Oncology Department, Harbin Medical University Cancer Hospital, Heilongjiang; b Department of Translational Medicine, Genetron Health (Beijing) Technology, Co. Ltd., Beijing, China.

**Keywords:** human epidermal growth factor receptor 2-positive metastatic breast cancer, multiple metastases, pyrotinib, rare mutations, under control

## Abstract

**Introduction:**

In about 15% to 20% of breast cancer cases, human epidermal growth factor receptor 2 (HER2) over-expression or gene-amplification is associated with poor prognosis. Thanks to the development of target therapies, HER2 positive patients can be managed using HER2-targeting drugs. There are several kinds ofHER2 inhibitors, such as trastuzumab, lapatinib, and pyrotinib. Pyrotinib which exert different functions, of note, the latest generation of the drug, is an irreversible small-molecule tyrosine kinase inhibitor targeting epidermal growth factor receptor (EGFR) (HER1) and/or HER2 and/or HER4. Both lapatinib and pyrotinib potentially target EGFR and/or HER2, but in some instances, induces different responses of patients with *EGFR* and/or *HER2* mutations. This is attributed to the different mutations in *EGFR* and *HER2* genes, which may form distinct types of HER2 dimers, with different binding capacities to drugs.

**Patient concerns:**

Five years ago, a patient underwent a radical mastectomy in an external hospital. Results of the resection histopathology revealed an invasive ductal carcinoma, pT3N0M0, stage IIB, HER2 positive. The lady patient received 6 cycles of adjuvant chemotherapy and was subjected to adjuvant trastuzumab therapy for 1 year. After a regular 1-year follow-up and in March 2018, she complained of chest pain and visited our hospital. We diagnosed her with metastatic breast cancer, positive for HER2.

**Diagnosis:**

positron emission tomography/computed tomography showed multiple metastases in the lung and sternum, while the breast lesions did not progress, the curative effect of which we evaluated as a progressive disease. Then, lapatinib integrated with chemotherapy was administered to the patient. After 5 cycles of the treatment, the patient experienced lower back pain. Through CT examination, it was revealed that she had multiple metastases in the lung and sternum, in addition to new metastases in the lumbar spine and right lobe of the liver. Moreover, magnetic resonance imaging revealed multiple metastases in the brain, and the disease further progressed. The results of circulating tumor DNA assays showed that other than *HER2* amplification, novel *EGFR*-*ZNF*880 fusion and *EGFR* E114K mutations developed.

**Interventions:**

The patient was administered with a combination of pyrotinib with chemotherapy.

**Outcomes:**

After 2 months of pyrotinib treatment, the metastases of the lung, sternum, lumbar spine, and right lobe of the liver disappeared. Also, the size of the brain metastases reduced while bone metastases were relieved. The curative effect was evaluated as a partial response. Following the results of circulating tumor DNA assays, *HER2* amplification, *EGFR*-*ZNF*880 fusion, and *EGFR* E114K mutations disappeared. However, since a small lesion was present in the brain, the patient was subjected to radiotherapy in the head. Notably, after 9 months treatment with pyrotinib, enhanced CT indicated that tumors in the breast, liver, both lungs, brain, and bone were under control. The patient continually received oral pyrotinib, however, a new brain lesion appeared 6 months later. Overall, we managed to regulate the efficacy of pyrotinib for up to 15 months.

**Conclusion:**

This case report demonstrates that *EGFR*-*ZNF*880 fusion and *EGFR* E114K mutations may contribute or lead to the formation of a special HER2 dimer, which is rapidly resistant to lapatinib but sensitive to pyrotinib. Of note, this is the first report that such a new fusion has been found.

## 1 Introduction

human epidermal growth factor receptor 2 (HER2) positive breast cancer accounts for 15% to 20% of all breast cancer cases.^[1]^ Compared with other subtypes, HER2 positive breast cancer is characterized by a poorer prognosis and predispose patients to brain metastasis.^[2]^ Management of HER2 positive patients using HER2 inhibitors has greatly improved the overall prognosis. Commonly used HER2 inhibitors include trastuzumab, patuzumab, lapatinib, pyrotinib, among others.^[3]^ However, HER2 positive metastatic breast cancer progressively develop resistance to trastuzumab, and some of the HER2 overexpressed breast cancer receiving trastuzumab-adjuvant therapy eventually relapse. Lapatinib is a small tyrosine kinase inhibitor targeting epidermal growth factor receptor (EGFR) and/or HER2 without any cross-resistance to trastuzumab. A combination of lapatinib with capecitabine has been proved to be effective in the treatment of HER2 overexpression breast cancer, which progresses post trastuzumab treatment.^[4]^ On the other hand, pyrotinib is an irreversible pan ERBB receptor tyrosine kinase inhibitor, which simultaneously targets EGFR and/or HER2 and/or HER4.^[5]^

Although both lapatinib and pyrotinib target EGFR/HER2, lapatinib and pyrotinib show varying effects in patients with *EGFR*/*HER2* mutation.^[6]^ In a previous study, 128 eligible patients were randomly assigned to the pyrotinib (n = 65) or lapatinib (n = 63) treatment groups, notably, the overall response rate was 78.5% (95% CI, 68.5% to 88.5%) with pyrotinib and 57.1% (95% CI, 44.9% to 69.4%) with lapatinib (treatment difference, 21.3%; 95% CI, 4.0% to 38.7%; *P* = .01). Besides, the median progression-free survival was 18.1 months with pyrotinib (95% CI, 13.9 months to not reached) and 7.0 months with lapatinib (95% CI, 5.6 to 9.8 months, adjusted hazard ratio, 0.36; 95% CI, 0.23 to 0.58; *P* < .001).^[7]^ This could be associated with the different mechanisms of lapatinib and pyrotinib. For instance, lapatinib attaches to ATP binding sites in the intracellular domain of EGFR/HER2, forming a slightly reversible inactive structure;^[8]^ Whereas, pyrotinib covalently attaches to ATP binding sites in the intracellular kinase region of EGFR/HER2/HER4, and irreversibly coordinates the formation of her family homologous/heterodimers. Consequently, pyrotinib inhibits autophosphorylation, blocks the activation of downstream signaling pathways, and inhibits tumor cell growth.^[9]^ Clinically, *EGFR/HER2* occurs in various mutation types, which may influence the formation of different EGFR/HER2 dimers, inducing different responses to lapatinib or pyrotinib.

In recent years, with the advancement in tumor molecular biology and the establishment of the “precision medicine” concept, breast cancer management is becoming more and more individualized. Gene testing could facilitate an in-depth understanding of the mechanisms of drug resistance and the selection of more sensitive drugs in ensuring that patients receive accurate clinical benefits. Herein, we report a case of *HER2* amplification metastatic breast cancer who developed novel *EGFR*-*ZNF*880 fusion and *EGFR* E114K mutations post lapatinib treatment but was successfully managed using pyrotinib for about 15 months, after which the mutation combo disappeared simultaneously. To our knowledge, this is the first report of *EGFR* fusion and mutation associated with lapatinib resistance and is characterized by sensitivity to pyrotinib.

## 2 Case presentation

**Figure 1:**
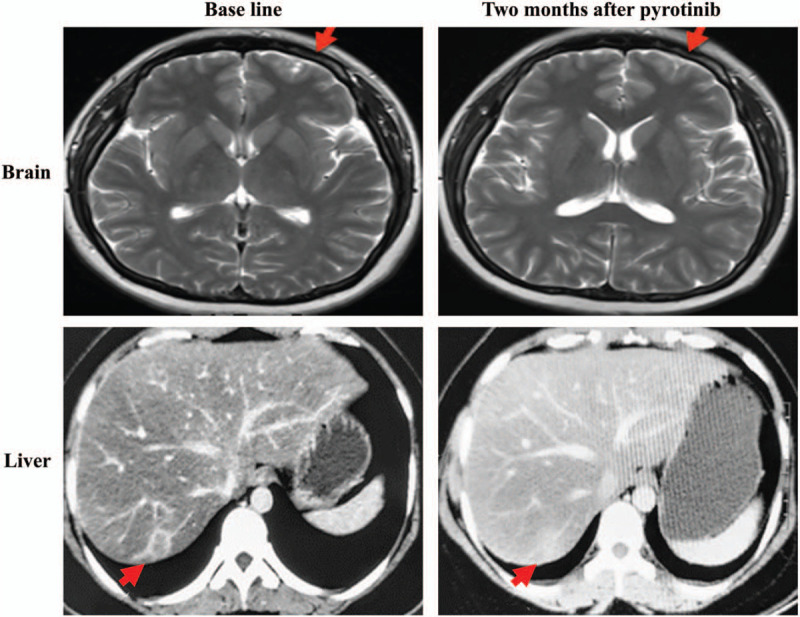
The magnetic resonance imaging of the brain and the computed tomography images (CT) of the liver before and after pyrotinib treatment. Red arrows represent the tumors.

**Figure 2:**
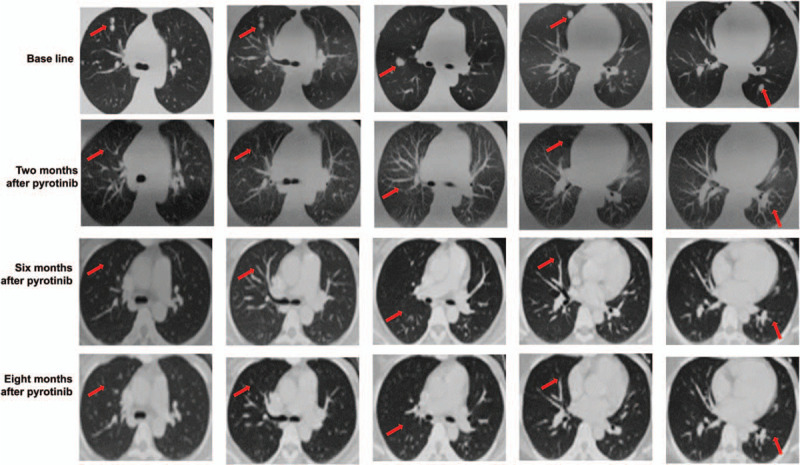
The computed tomography images (CT) of the lung before and after pyrotinib treatment. Red arrows represent the tumors.

We assessed a 34-year-old female patient without a family history of breast cancer. Five years ago, on June 30, 2015, she underwent left breast radical mastectomy in an external hospital. The postoperative pathological diagnosis was invasive ductal carcinoma of the left breast, T3N0M0, stage IIB, with a tumor size of 6.5 × 6.5 × 3.8 cm. Immunohistochemistry analysis of the tumor resection showed positive results for ERBB2, partial positive results for Ki-67 (35%) and p53 (< 1%), and negative results for ER and PR. Additionally, FISH testing revealed positive amplification of HER2 (HER2/CEP17 = 5.167), an interpretation which was based on the criteria of HER2 double probe in situ hybridization in the 2013 ASCO/CAP guidelines. Docetaxel (75 mg/m2, D1) plus doxorubicin liposome (60 mg/m^2^, D1) were administered to the patient for 6 cycles. At the same time, the patient was treated by trastuzumab (Loading 8 mg/kg, then 6 mg/kg, d1, 1/21d) for one year. After regular follow-up for one year, the patient complained of chest pain, and we admitted her to our hospital for treatment in March 2018. Her ECOG score was 0 to 1 at that time. Positron emission tomography/computed tomography showed multiple metastases in both lungs, lymph node metastasis in the left internal mammary gland and right hilum, and bone metastases in the sternum and left iliac bone. No definite abnormality was identified through the head enhanced magnetic resonance imaging. Following the response evaluation criteria in solid tumors, version 1.1 (RECIST v1.1) that new lesions are identified as a progressive disease, the patient exhibited a progressing disease at this time. The patient underwent subsequent treatment with 5 cycles of lapatinib (1250 mg/d) plus Xeloda (1.5 g, bid, d1-14, 1/21d) in addition to other combination strategies, for example, treatment of anti-bone metastasis. However, at the end of the treatment, the patient was immediately admitted to our hospital with low back pain. Brain magnetic resonance imaging showed intracranial metastatic tumors (Fig. 1; baseline), whereas a CT scan showed new lesions in the right lobe of the liver (Fig. 1, baseline) and multiple metastases of the lung (Fig. 2, baseline). According to the RECIST v1.1 standard, the patient had a progressing disease. At this time, other than HER2 amplification, two novel mutation, *EGFR*-*ZNF*880 fusion and *EGFR* E114K mutations, were identified in circulating tumor DNA by NGS. As aforementioned, previous investigations proved that either EGFR or HER2 mutations could influence the formation of HER2 dimers, whereas lapatinib and pyrotinib potentially induced responses to different HER2 dimers. Thus, the combination strategy of pyrotinib (400 mg/d), albumin paclitaxel (125 mg/m2, d1, d8, 1/21d∗6) and carboplatin (AUC6, d1, 1/21d∗6) was performed. After 2 months of treatment, the brain lesions shrunk significantly (Fig. 1), liver and lung lesions disappeared (Fig. 1 and Fig. 2), and the bone metastasis was relieved. According to the RECIST v1.1 standard, the curative effect could be evaluated as a partial response. Following the genetic test of circulating tumor DNA, all mutations disappeared, including *HER2* amplification, *EGFR*-*ZNF*880 fusion, and *EGFR* E114K mutations. Due to the existence of brain lesions, the patient was subjected to brain radiotherapy at this time. After 9 months of pyrotinib treatment, enhanced CT showed diffuse changes in the liver, whereas no changes were evident in both lungs. The disease stabilized, thus the patient continued to receive oral pyrotinib. Unfortunately, the disease progressed by November 2019, whereby a novel brain lesion appeared. Overall, the efficacy of pyrotinib was regulated for 15 months. Similarly, the overall adverse reactions were manageable.

## 3 Discussion

Based on the current understanding, HER2 form dimers with other members of its family, such as EGFR and HER3, which potentially induce HER2 autophosphorylation of tyrosine kinase region in the intracellular domain, initiate intracellular signal cascade reaction, maintain cell survival and development, and promote cell proliferation and migration.^[10]^ Simultaneous mutation of HER2 and other EGFR family members may result in the failure of trastuzumab or lapatinib to block the interaction among her family members, causing drug resistance. As previously reported, *EGFR* V843I mutation is one of the mechanisms associated with lapatinib resistance.^[11]^ Here this case also proved similar resistance mechanism of lapatinib. In this case study, the patient previously received anthracycline, taxanes, trastuzumab adjuvant therapy after surgery. When relapse, lapatinib combined with chemotherapy was used to manage the patient. However, no remission in the lung and bone metastases was reported; novel metastases developed; *HER2* amplification existed; novel mutations of *EGFR*-*ZNF*880 fusion and *EGFR* E114K were detected. Notably, when the patient was treated with pyrrolidine combined with chemotherapy, liver and lung lesions disappeared. *HER2* amplification, *EGFR*-*ZNF*880 fusion and *EGFR* E114K mutations disappeared. The *HER2* amplification, the fusion of *EGFR*-*ZNF*880, and mutation of *EGFR* reported, in this case, may have led to the formation of a specific heterodimer between EGFR (HER1) and HER2, which was sensitive only to pyrotinib but ineffective against lapatinib. Thus, we concluded that the activated form of HER2 dimer is a crucial factor that drives the resistant mechanism of lapatinib. These findings suggest that clinicians should consider the formation pattern of HER2 dimers in HER2 positive patients, especially the dimers that are potentially associated with the mutations of HER family numbers (e.g., *EGFR*-*ZNF880* fusion and *EGFR* E114K mutations). When lapatinib fails, pyrotinib therapy could be considered.

Furthermore, *EGFR*-*ZNF*880 fusion is a novel mutation that has yet to be included in COSMIC (COSMIC, Aug2018), and no function analyses were reported; *EGFR* E114K mutation, which has been reported in breast cancer and other cancers (COSMIC, Aug2018), though its function remains elusive. Limited information has currently been published about both mutations, which may affect our understanding of the resistance mechanism of lapatinib. Therefore, future investigations should purpose to elucidate the precise resistance mechanism of HER2 inhibitors extensively.

## 4 Consent for publication

We have obtained written informed consent from the patient for publication of this case report and accompanying images.

## Author contributions

**Conceptualization:** Liu Wentao, Fu Na.

**Data curation:** Huang Jingjing.

**Edited the original manuscript and revised the manuscript following peer-review:** Tonghui Ma, Dalin Li.

**The attending doctors of the patient:** Wentao Liu, Na fu.

**Writing – original draft:** Zhang Xin.

**Writing – review & editing:** Li Yue, Zhang Xin, Zhang Xiaoyan, Ma Tonghui, Li Dalin.

**Writing – performed the procedure & edited the manuscript:** Xiaoyan Zhang, Jingjing Huang.

**Writing – original draft & information collection:** Yue Li, Xin Zhang.
